# Correction: Prescribing patterns and associated factors of antibiotic prescription in primary health care facilities of Kumbo East and Kumbo West Health Districts, North West Cameroon

**DOI:** 10.1371/journal.pone.0196861

**Published:** 2018-04-30

**Authors:** The PLOS ONE Staff

## Notice of republication

This article was republished on April 23, 2018, as the original Supporting Information file S1 Text was published in error. The publisher apologizes for the error. Please download this article again to view the correct version. The originally published, uncorrected article and the republished, corrected article are provided here for reference.

## Supporting information

S1 File Originally published, uncorrected article. (PDF)

S2 File Republished corrected article. (PDF)
