# Supplementary material for: Prescribing patterns and associated factors of antibiotic prescription in primary health care facilities of Kumbo East and Kumbo West Health Districts, North West Cameroon
Source: PLoS One. 2018 Mar 5;13(3):e0193353. doi: 10.1371/journal.pone.0193353 (PMC5837085; doi:10.1371/journal.pone.0193353)
Supplement: S1 Text — (DOCX) [file pone.0193353.s001.docx]

**QUESTIONNAIRE ADMINISTERED TO PRIMARY HEALTH CARE PERSONNEL PRESCRIBING ANTIBIOTICS IN KUMBO EAST AND WEST HEALTH DISTRICTS OF THE NORTH WEST REGION.**

Health District Health Area Health Facility

Code

Presence of Essential Drug List: Yes  No

**DEMOGRAPHIC INFORMATION**

1. Age:
2. Sex: Male  Female
3. Professional level: Nurse Ass  Brevete Nurse  Midwife  HND  SRN  BSN  GP  PG  specialty
4. Duration in practice

**A. PRESCRIBER FACTORS**

1. How long have you been prescribing antibiotics?(Longevity):
2. Does the availability of antibiotics motivate you to prescribe antibiotics frequently? Yes  No
3. How often do you prescribe antibiotics for prophylactic purposes?

Not at all (0 times) rarely/sometimes (once a month)  often (weekly)  very often (daily)  others (specify)

1. Does the presence of fever automatically influence you to prescribe antibiotics? Yes  No  Do not Know
2. Do you follow any treatment guideline before prescribing antibiotics? Yes  No
3. If yes, specify
4. Do you use Laboratory guidance (culture and sensitivity) before antibiotic prescription? Yes  No  Do not Know
5. Are you aware of the existence of Antibiotic Resistance? Yes No
6. Do you update your knowledge on prescription practices? Yes  No
7. How often? Daily  Weekly  Monthly  Yearly  other  (specify)
8. Method of updating knowledge on clinical medicine and prescription practices:

Internet resources  Textbooks  Conferences  Refresher courses  Peer review meetings  others  (specify):

1. Does the Patient turnout per day affect your prescription decision? Yes  No
2. Do you at times prescribe specific antibiotics based on observed treatment success rate of that particular antibiotic in the field and not based on laboratory findings? Yes  No
3. **INSTITUTIONAL FACTORS**
4. Are there institutional regulations/policies that determine your choice and rate of prescribing antibiotics? Yes  No
5. If yes specify
6. Are your prescriptions subject to evaluation from any regulatory body (internal or external)? Yes  No  Do not Know
7. If yes specify
8. Others institutional factors if any:
9. In your own opinion, what do you think should be done to improve antibiotic prescribing practices?

Thanks for your collaboration
