# Supplementary material for: Correction: Prescribing patterns and associated factors of antibiotic prescription in primary health care facilities of Kumbo East and Kumbo West Health Districts, North West Cameroon
Source: PLoS One. 2018 Apr 30;13(4):e0196861. doi: 10.1371/journal.pone.0196861 (PMC5927450; doi:10.1371/journal.pone.0196861)
Supplement: S1 File — (PDF) [file pone.0196861.s001.pdf]

PLOS ONE

RESEARCH ARTICLE

Prescribing patterns and associated factors of antibiotic prescription in primary health care facilities of Kumbo East and Kumbo West Health Districts, North West Cameroon

Elvis Dzelamonyuy Chem, Damian Nota Anong, Jane-Francis K. T. Akoachere*

Department of Microbiology and Parasitology, Faculty of Science, University of Buea, Buea, Cameroon

* jakoachere@yahoo.com

Abstract

Background

Inappropriate use of antibiotics is a global public health challenge and has been associated with antibiotic resistance. WHO reports show that efforts to promote rational antibiotic use in developing countries are poor. With the growing number of infections with antibiotic resistant bacteria, rational drug use becomes imperative and studies that promote rational drug use are highly necessary. Considering this, we investigated prescribing patterns and predictors of antibiotic prescription in primary health care facilities in Kumbo East (KE) and Kumbo West (KW) health districts in North West Cameroon, to contribute data which could influence policy on antibiotic use.

Methods and findings

A cross sectional retrospective study was conducted from April 2014 to April 2015 in 26 randomly selected primary care facilities. Questionnaires were administered to 59 antibiotic prescribers to determine factors that predict antibiotic prescribing. Data on antibiotic prescription were collected by review of consultation registers. Prescription rates and demographics, prescriber and institution factors were analyzed using ANOVA. The best predictor of prescription was determined using multiple linear regression analysis.

Results

A total of 30,096 prescriptions were reviewed. Overall antibiotic prescription rate was 36.71%, with a mean of 1.14 antibiotics prescribed per patient. Amoxicillin was the most prescribed (29.9%). The most prevalent indications for prescribing were respiratory tract infections (21.27%). All antibiotics prescribed were broad-spectrum. Antibiotics were prescribed for patients with malaria and also in situations where diagnosis was uncertain. Prescribing by generic name was 98.36% while 99.87% was from Essential Drug List. Use of laboratory results, patient turnout and Performance Based Financing (PBF) were significantly associated with antibiotic prescribing rates ($p < 0.05$). PBF moderated prescribing.

OPEN ACCESS

Citation: Chem ED, Anong DN, Akoachere J-FKT (2018) Prescribing patterns and associated factors of antibiotic prescription in primary health care facilities of Kumbo East and Kumbo West Health Districts, North West Cameroon. PLoS ONE 13(3): e0193353. https://doi.org/10.1371/journal.pone.0193353

Editor: Brian Godman, Karolinska Institutet, SWEDEN

Received: December 1, 2016

Accepted: January 31, 2018

Published: March 5, 2018

Copyright: © 2018 Chem et al. This is an open access article distributed under the terms of the Creative Commons Attribution License, which permits unrestricted use, distribution, and reproduction in any medium, provided the original author and source are credited.

Data Availability Statement: All relevant data are within the paper.

Funding: This study was sponsored by the investigators.

Competing interests: The authors have declared that no competing interests exist.

Conclusion

There was misuse of antibiotics in primary care facilities in study area. We recommend all primary care health facilities in study area to be included in the PBF scheme and that prescribing should only be done by physicians as the have adequate training.

Introduction

Antimicrobial drugs have been widely used in human medicine for more than 50 years either as prophylaxis or therapeutics, with tremendous benefits to human health. Unfortunately, widespread use, misuse or inappropriate prescribing has resulted in the emergence of drug resistant bacteria [1]. Antibiotic resistance is a global public health concern. Studies [2] have reported a positive relationship between antibiotic utilization and the level of antibiotic resistance. The number of infections due to antibiotic-resistant bacteria is growing and outpacing the rate at which new classes of antibiotics are discovered and synthesized [3]. Antimicrobial resistance is also a barrier to public health efforts in the control of infectious diseases through specific disease control programmes that rely on the use of antimicrobials as a strategy for control and prevention. Prudent use of antimicrobials helps to prevent the relentless increase in resistance.

Infections with drug-resistant bacteria have increased not only morbidity and mortality but also duration of hospitalization and cost of treatment. When infections become resistant to first-line antibiotics, more expensive second-line therapies must be used, resulting in a longer duration of illness and treatment in hospitals which often increases health care costs as well as the economic burden on families and societies [4, 5] as the intensity of care needed by patients with infections caused by drug resistant bacteria is different from that in patients with infections caused by drug sensitive bacteria. Because the human economic cost of playing catch up on antibiotic resistance is too great a risk to bear, there is need for expansion of research and surveillance beyond developed countries to adequately control antibiotic resistance [6].Current estimates of economic burden due to antimicrobial resistance in the United Kingdom range from less than 5 GB£ to more than 20,000 GB£ in reported additional costs per patient for each episode of illness in hospital costs [7]. A study in Thailand [8] reported hospitalization cost of US$ 528 in patients with community-onset extended spectrum beta lactamase-producing *E. coli* infections compared to US$ 108 in those with community onset non-extended spectrum beta lactamse-producing *E. coli* infections.

Field surveys carried out in resource poor countries have highlighted a significant degree of inappropriate use of drugs in health facilities as well as in the community and this has gone a long way to add to the ever increasing cost of health care delivery especially when drug resistance ensues [9,10]. According to WHO reports, less than 40% and 30% of patients in public and private facilities respectively are treated under WHO guidelines [11]. To ensure rational drug prescription, prescribers must adhere to treatment guidelines and must also follow a standard process of prescribing [12]. Regrettably, prescribers often use broad spectrum antibiotics to manage suspected cases of both gram positive and gram negative bacterial infections [13] and in some instances, antibiotics are prescribed for conditions not requiring antibiotic treatment [14]. Drug prescription patterns therefore have a great impact on the outcome of patient's conditions, and hence have to be evaluated periodically so that interventions can be implemented if necessary, to ensure prudent drug use. To evaluate the situation of antibiotic use and prescription patterns in the field, the WHO has developed prescribing indicators for health care facilities [15].

A variety of factors have been found to influence (predict) antibiotic prescription. These include patient characteristics such as low-socioeconomic status, age of patient and co-morbidity; physician factors such as educational qualification, experience of the physician, source of updating knowledge; and practice setting [16–21]. Other important factors identified by doctors that influenced antibiotic prescription are; diagnostic uncertainty, perceived demand and expectation from the patients, practice sustainability, influence from medical representatives and inadequate knowledge [22–24]. In a study carried out in Nigeria, drug availability, socio-economic status of the patient and prescriber in-service training were identified as major factors influencing prescription decisions [25]. It is necessary to understand physicians prescribing behavior in order to develop interventions that will effectively improve the use of antibiotics.

About 80% of antibiotic use occurs in the community, with the bulk of it contributed by either primary health care providers or self-medication [26]. Primary health care facilities are dedicated to improving the health status of the community and act as the first line of contact with the community, for acceptable, accessible and affordable care. Community based health system strengthening programs that improve health seeking behavior and transform health care services such as the performance-based financing program, are more evident at the primary health care level [27,28]. Consequently, the that majority of the population in communities in a developing country like Cameroon will revert to primary health care facilities for immediate care considering the accessibility and affordability of the services provided. Also, the limited number of secondary care facilities and high cost of health care (which is usually paid out of pocket) automatically diverts the choice of facility to primary care, for majority of the population who live on less than one dollar per day. Thus, the majority of antibiotic prescribing takes place in the primary health care setting.

Primary care is a major contributor to antibiotic resistance. Studies have provided strong evidence of an association between the prescribing of antibiotics in primary care and antimicrobial resistance [1,29,30]. To combat antibiotic resistance, interventions on inappropriate antibiotic use must be implemented and interventions are more effective if they are multifaceted [1,12] and include improved communication between healthcare providers and patients [31]. These initiatives target prescribing practices by clinicians or self-medication by patients as self- medication is rife in Africa [32–34]. For these interventions to be successfully implemented, data on antibiotic prescription and associated factors are necessary.

Antibiotic prescription and treatment in resource poor settings including Cameroon is mostly empiric due to the high cost of laboratory investigations, lack of facilities for culture and antibiotic sensitivity testing, and the long duration required for reporting results of laboratory investigations. In addition, a dearth of knowledge on antibiotics prescription and factors influencing prescription exists in Cameroon. Mbam *et al.* [14] attributed inappropriate prescribing of antibiotic in Buea Regional hospital, a secondary care facility, to lack of clearly defined working diagnosis and identified polypharmacy as a problem in this facility. With the growing reports on antibiotic resistance in Cameroon [35–37] there is need to investigate drug use patterns. Consequently, our study investigated prescribing patterns and associated factors of antibiotic prescribing in primary health care facilities in Kumbo East (KE) and Kumbo West (KW) health districts in the North West region of Cameroon. As a result, contribute data which can influence future policiesto improve antibiotic use in Cameroon.

Materials and methods

Study setting and design

This study was carried out in Kumbo East and Kumbo West health districts in Bui Division, North West region of Cameroon. Kumbo East and Kumbo West are two of the 19 health

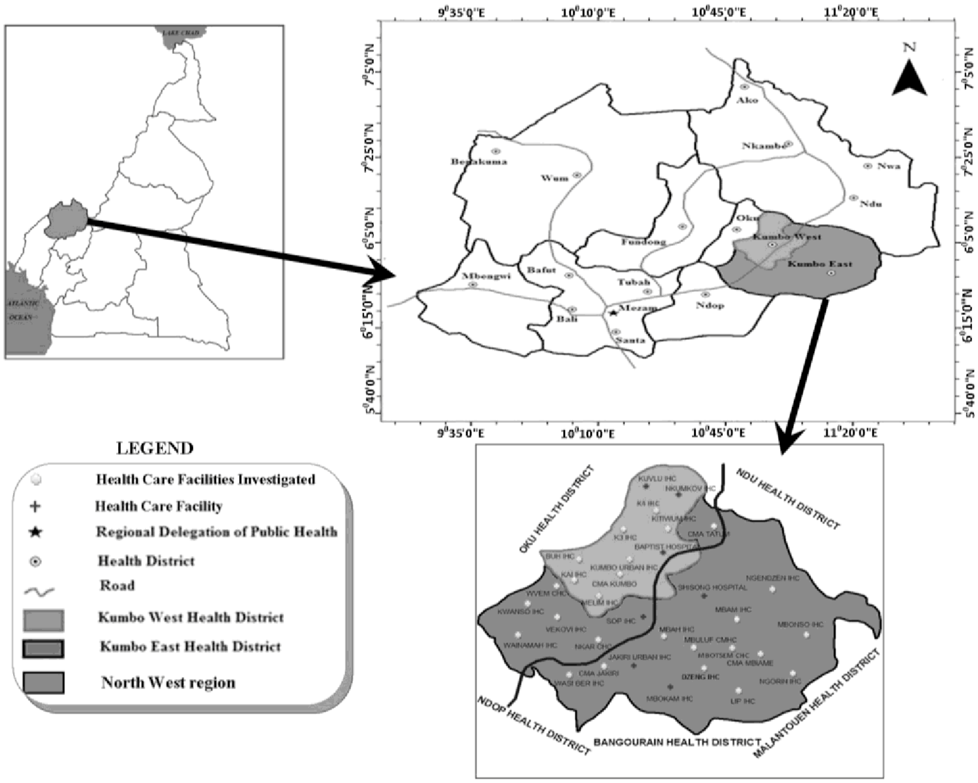

Fig 1. Map of study area.

https://doi.org/10.1371/journal.pone.0193353.g001

districts in North West region. They cover a surface area of 2124 km^2 and are largely rural. Kumbo West (KW) is bounded to the west by Oku health district and to the east and south by the Kumbo East (KE) health district (Fig 1). K E is bounded to the north by Ndu Health district, to the east by the Malantouen health district in the West region, and to the south by Ndop and Bangourain health districts (Fig 1). These two health districts harbor most of the primary health care facilities in the North West region of Cameroon because in addition to public facilities, they have several confessional primary care facilities established by the Baptist and Catholic Missions. Because of this, there are more consultations at primary care facilities in these health districts. Consequently, chosen for this project, according to 2012 estimates, KE has a population of about 171,126 [38] with 30 primary health care units (16 public, 7 confessional, 4 community and 3 private), while KW has 19 primary health care units (10 public, 6 confessional, 2 community and 1 private) and a population of 96,829. Each primary health care unit provides a minimum package of health activities to the population under care, including consultation and essential drug prescription.

Data on antibiotic prescription in health facilities studied was collected by review of consultation registers.

Questionnaires

Questionnaires that had been piloted and validated were administered to health care personnel who prescribed antibiotics from April 2014 to April 2015 to determine the factors that influence antibiotic prescribing. The questionnaire had three sections: demographic information of the prescriber, prescriber related factors that influence prescribing and institutional factors that might influence prescribing (S1 Text).

Review of consultation register

Demographic data (age, sex) of patient, use of laboratory guidance (laboratory request and use of laboratory results to guide prescription), disease diagnosed and the type and number of antibiotics prescribed for the diagnoses were collected for each patient who consulted. The total number of prescriptions by each prescriber from April 2014 to April 2015 was counted and the percentage with antibiotics calculated. The total number of antibiotic prescriptions in this study was obtained by summing the number of prescriptions for each prescriber. The performance of health care providers in relation to antibiotic use was measured using WHO indicators of prescribing [15]. The average number of antibiotics prescribed per patient prescription was determined, the prescription rate per 100 prescriptions calculated and the percent of encounters with two or more antibiotics analyzed. Other drug prescribing indicators such as; % of antibiotics prescribed by generic name, % encounters with an antibiotic prescribed, % of antibiotics prescribed from essential drug list (EDL) and % of facilities with a copy of EDL, were also investigated. Four trained research assistants assisted in data collection.

Sampling technique

A cross sectional and retrospective study was conducted between April 2014 and April 2015. Questionnaires were administered to 59 antibiotic prescribers from 26 randomly selected primary health care facilities to determine factors that could predict antibiotic prescribing. A multi-stage, stratified sampling (between KE and KW health districts) was done, with further stratification of primary health care facilities in each health district into low, medium or high population primary health facilities based on their population under care/health area population. Clustered random sampling was used to select participating facilities from each of the final stratum.

For stratified sampling at the district level, all primary health care facilities were primarily stratified into KE and KW health facilities. Within each district, these facilities were further stratified into three groups according to the size of the population under care into; low (<5000 inhabitants), moderate (between 5000 to 10000 inhabitants) and high (> 10000 inhabitants).

After stratification, a randomized clustered sampling was done to select the participating health care facilities from each stratum. At the practice level, a census of prescribers within each participating facility was done and all prescribers targeted.

Ethical considerations

Ethical clearance for this study was obtained from the Institutional Review Board of the Faculty of Health Sciences, University of Buea. Administrative clearance was obtained from the District Medical Officers of Kumbo East and Kumbo West and also from the management of health care facilities. All participants were informed about the objectives of the study and their enrollment in the study was voluntary. Prescribers were also informed on free withdrawal at any time. Confidentiality of the results was maintained by using codes in place of prescriber names in the questionnaires. Study participants signed an informed consent form to indicate their willingness to participate in the study.

Data analysis

The statistical package EPI Info 7 was used to design the questionnaire template and to enter data in this study. SPSS version 20.0 statistical software was used in data cleaning, management and analysis. A descriptive analysis on the cases was done. The relationship between antibiotic prescribing rates (the dependent variable) and the independent variables (demographic

factors/prescriber/institution related factors) was analyzed using analysis of variance and correlation analysis. Multiple linear regression analysis was used to determine the best associated factor of prescription from several independent variables.

Results

Characteristics of prescribers and patients prescribed antibiotics

Of the 59 prescribers who received the questionnaire, 56 completed it giving a response rate of 95%. Prescribers who participated in the study comprised 47 (83.9%) females and 9(16.1%) males. The majority (41.1%) of the prescribers were aged 30–39 years, while 25% and 17.9% of the population were 40–49 years and 20–29 years respectively (mean age: 38.04 ± 9.28.3SD, range: 24–60 years). Nurse assistants (42.8%) comprised the majority of prescribers, followed by State Registered Nurses (25%) and General Practitioners (8.9%), while Medical Assistant (1.8%) and Nurses with Higher National Diploma (HND) (1.8%) were the least prescribers (Table 1). There was no significant difference among prescribers with respect to gender (F = 0.084, P = 0.773), age (R = 0.0010, P = 0.943) and profession (F = -0.907, P = 0.369). Most of the prescribers were from public facilities (69.6%) followed by those from confessional facilities (26.8%).

For the patients whose records were reviewed, 38.34% were male and 61.7% females and age ranging from 8 months to 90+ years. Among these, 35.2% of males and 37.6% of females received antibiotics (Table 2). The age group most subjected to antibiotics were those <10years (44%). This was followed by those 21–30 years (40%). There was a significant

Table 1. Characteristics of prescribers.

Characteristic	Categories	n (%)	P value
**Gender**	Male	9 (16.1)	F = 0.084. P = 0.773
	Female	47 (83.9)	
	**Total**	**56**	
**Age (years)**	< 20	0 (0)	R = 0.010, P = 0.943
	20–29	10 (17.9)	
	30–39	23 (41)	
	40–49	14 (25)	
	> 50	9 (16.1)	
	**Total**	**56**	
**Professional level (Qualification)**	Medical Assistant	1 (1.8)	F = -0.907, P = 0.369
	Nurse Assistant	27 (42.8)	
	Brevete Nurse	4 (7.14)	
	Higher National Diploma (HND) in Nursing	1 (1.8)	
	State Registered Nurse	14 (25)	
	Bachelor of Science in Nursing	2 (3.6)	
	General Practitioner	5 (8.9)	
	Midwives	2 (3.6)	
	**Total**	**56**	
**Type of Facility**	Public	39 (69.6)	F = 0.99, P = 0.921
	Private	1 (1.8)	
	Confessional	15 (26.8)	
	Community	1 (1.8)	
	**Total**	**56**	

https://doi.org/10.1371/journal.pone.0193353.t001

Table 2. Characteristics of patients prescribed antibiotics.

Characteristic	Categories	Number participated (%)	Number that received antibiotic prescription	Percentage that received antibiotics	P value
**Gender**	Male	11530 (38.3)	4061	35.2	F = 8.94, P = 0.000
	Female	18566 (61.7)	6974	37.6	
	**Total**	30096 (100)	11035		
**Age (years)**	≤ 10	10534 (35)	4597	44	F = 3.40, P = 0.005
	11–20	4665 (15.5)	1492	32	
	21–30	5055 (16.8)	1997	40	
	31–40	3250 (10.8).	1157	36	
	41–50	2469 (8.2)	791	32	
	51–60	2037 (6.8)	512	25	
	61–70	1171 (3.9)	334	29	
	71–80	513 (1.7)	108	21	
	81–90	332 (1.1)	33	10	
	> 90	71 (0.2)	14	20	
**Total**		30096 (100)	11035	36.71	

https://doi.org/10.1371/journal.pone.0193353.t002

difference in antibiotic exposure with respect to sex (F = 8.94, P = 0.000) and age (F = 3.40, P = 0.005).

Antibiotic prescription rate

A total of 30,096 prescriptions were reviewed from consultation registers in the 26 primary health care facilities selected for this study. 11,035 of them had at least one antibiotic prescription (Fig 2) giving an overall percentage prescription with antibiotics of 36.7%. Because some prescriptions had more than one antibiotic prescribed, a total of 12,350 antibiotics were prescribed during the study period (Fig 2). The majority of prescriptions (87.42%) had one antibiotic. This was followed by those with two antibiotics (11.54%). There were two prescriptions with up to 4 antibiotics (Fig 3).

Antibiotic prescription rates in various primary health care facilities are shown in Fig 4. There was no significant difference in the antibiotic prescription rate between KE and KW (H = 2.7003, P-0.100). The antibiotic prescription rate for prescribers ranged from 10.7–58.7%. In terms of facility, modal prescription class with high antibiotic prescription rates was 41–47%. Within the participating facilities, 18 prescribers constituted the class 42–51%, with high antibiotic prescription rates.

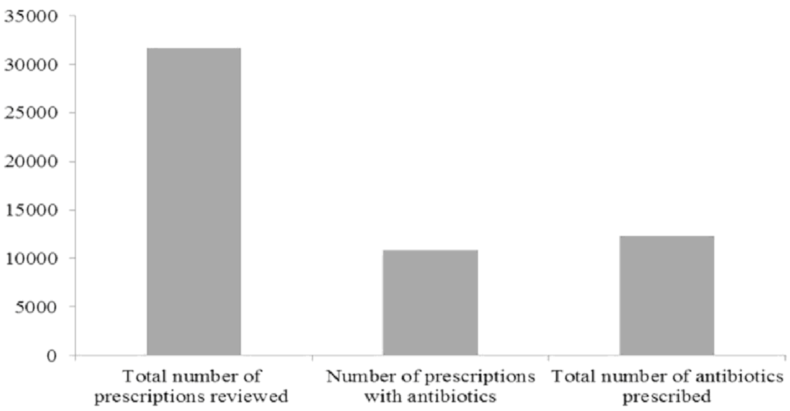

Fig 2. Number of prescriptions reviewed and antibiotic prescription rate.

https://doi.org/10.1371/journal.pone.0193353.g002

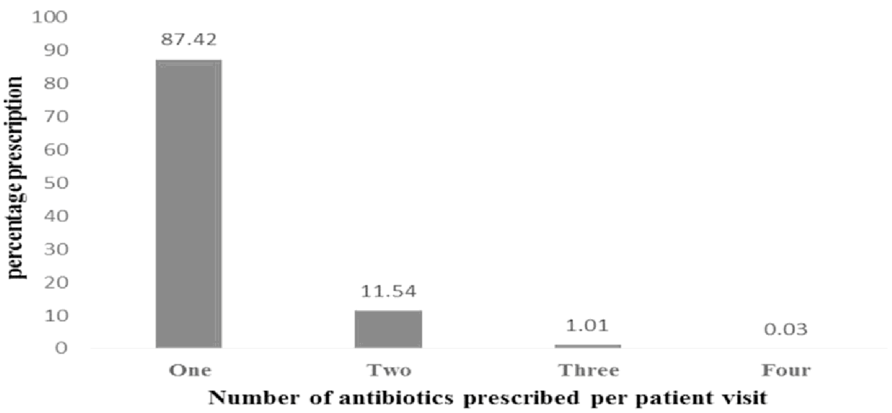

Fig 3. Number of antibiotics per prescription.

https://doi.org/10.1371/journal.pone.0193353.g003

Antibiotic prescribing pattern

The overall percentage of prescriptions with antibiotics was 36.71 (Table 3). Antibiotic prescription rate with regards to the type of health facility was as follows: low population health facilities (38.56%), > high (36.86%) > medium (34.67%) (Table 3). The average number of antibiotics prescribed per encounter in study area was 1.14.The percentage of antibiotics prescribed by generic name in all facilities was 98.36%, while prescriptions with brand names comprised 1.64% of all prescriptions. The overall percentage of drugs prescribed from EDL was 92.9 and 96.3% of all primary health care facilities had a copy of EDL. There was no statistically significant variation between low, medium and high level facilities with respect to: antibiotic rate, percentage of drugs prescribed by generic name and percentage of facilities with a copy of EDL ($P > 0.05$) (Table 3). Significant differences ($P<0.05$).were however observed between health facilities with regards to the number of antibiotics per prescription and % of drugs prescribed from the EDL.

Antibiotics prescribed and indications for prescription

The 12350 antibiotics prescribed during the study period belonged to 10 different classes. The majority (13/14, 92.9%) of these were broad spectrum (Table 4) and mostly the penicillins. The most prescribed antibiotics were Amoxicillin (29.29%) appearing 11.31 times per 100 prescriptions, Cotrimoxazole (19.08%) appearing 7.37 times per 100 prescriptions and Metronidazole (15.59%) 6.02 times per 100 prescriptions (Table 4).

A total of 21 different types of diagnosis subject to antibiotic prescription were observed. Respiratory tract infections (21.27%) was the most prevalent indication, followed by uncomplicated malaria (11.42%) and gastrointestinal tract infections (11.30%), while dysentery (0.27%) was the least (Fig 5). We also noted prescribing of antibiotics (5.76%) in situations where there was no indication for diagnosis. Such were recorded as "No diagnosis" (Fig 5).

Clinical and socio-demographic predictors of antibiotic prescribing

A multiple linear regression analysis was used to determine if the following factors- professional level, type of facility, longevity in service, use of treatment guidelines, knowledge update, use of laboratory results prior to prescribing, patient turnout, observed treatment success and performance based financing–were associated with antibiotic prescribing. Among these, use of laboratory results prior to prescription ($F = 4.320$, $P = 0.000$), patient turnout ($F = -3.317$,

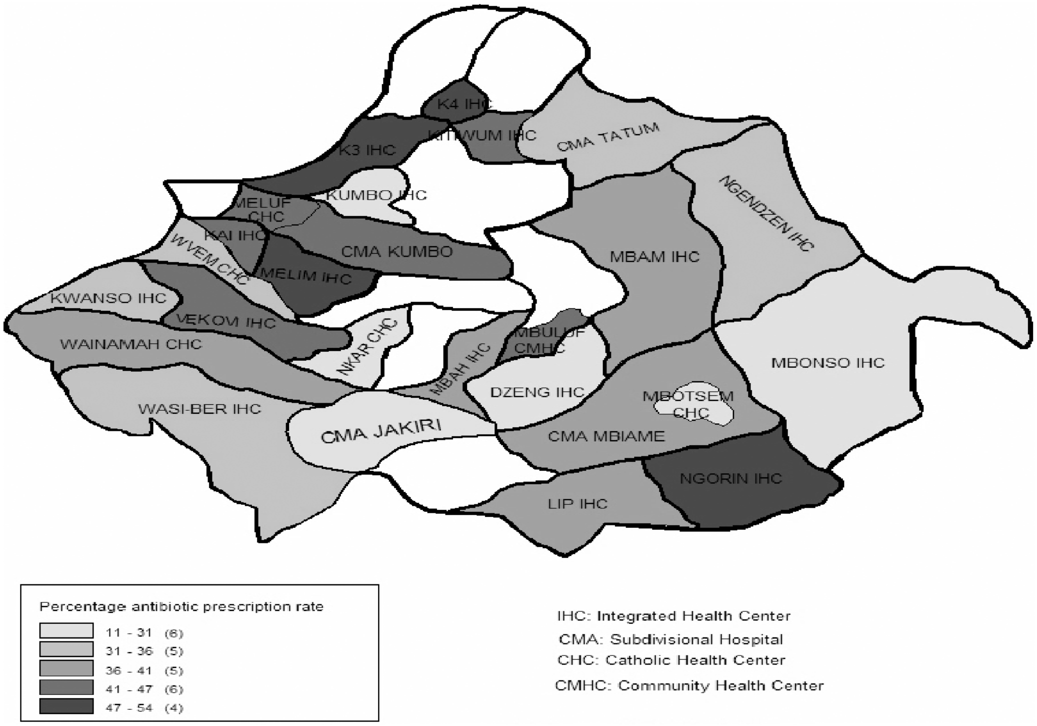

Fig 4. Antibiotic prescription rate in health care facilities in Kumbo East and Kumbo West health districts between April 2014 and April 2015.

https://doi.org/10.1371/journal.pone.0193353.g004

P = 0.002) and performance based financing health service program (F = 2.887, P = 0.006) were significant ($p < 0.05$) (Table 5).

Kruskall-Wallis H statistic and Spearman's correlation (R) were used to compare the antibiotic prescription patterns (rate of prescription of one, two and three antibiotics per patient encounter) with associated variables. Statistically significant variations ($P<0.05$) were observed with use of laboratory guides and patient turnout. Type of facility and longevity in service also significantly influenced the prescription of 2 and 1 antibiotics respectively (Table 5).

Performance Based Financing health service programme, grouped health care facilities into the following categories: T1 (Test centers 1), C1 (Control centers 1), C2 (Control centers 2) and C3 (Control centers 3).There was a significant variation (F = 2.887, P = 0.006) in observed antibiotic prescription rates between the facilities, with a systematic increase in prescription rates from T1, through C1, C2 and C3 facilities (Fig 6). Performance Based Financing was further identified as a moderating variable, influencing the outcome of other factors that could

Table 3. Antibiotic use indicators of primary health care facilities.

Antibiotic use indicator	Overall %	Primary health facility level			F value	P value
		Low	Medium	High		
Antibiotic prescription rate	36.71%	38.56%	34.67%	36.86%	0.044	0.957
Av. No of antibiotics per prescription	1.14	1.10	1.10	1.22	11.179	0.000
% of drugs prescribed by generic name	98.36	98,65	100	96.42	3.157	0.051
% of drugs prescribed from EDL	99.87	100	99.97	99.64	4.047	0.023
% of PHCF with a copy of EDL	96.3	88.9	100	100	1.5290	0.226

EDL: Essential Drug List; PHCF: Primary Health Care Facilities, Av: Average

https://doi.org/10.1371/journal.pone.0193353.t003

Table 4. Antibiotic category and prescription rates per 100 prescriptions.

Class	Antibiotic	Spectrum	No. of times Prescribed	Antibiotic prescription rate/100 prescriptions	Percentage prescription
Penicillin	Amoxicillin	Broad	3617	11.31	29.29
	Cloxacillin	Broad	1287	4.03	10.42
	Ampicillin	Broad	319	1.00	2.58
	Benzyl Pen	Broad	228	0.71	1.85
	Penicillin V	Broad	207	0.65	1.68
Antifolates	Cotrimoxazole	Broad	2356	7.37	19.08
Nitroimidazole	Metronidazole	Broad	1925	6.02	15.59
Quinolone	Ciprofloxacin	Broad	685	2.14	5.55
Aminoglycoside	Gentamycin	Broad	669	2.09	5.42
Cephalosporin	Ceftriaxone	Broad	387	1.21	3.13
Tetracycline	Doxycycline	Broad	368	1.15	2.98
Aminocycltol	Spectinomycin	Broad	13	0.04	0.11
Macrolide	Erythromycin	Broad	169	0.53	1.37
Chloramphenicol	Chloramphenicol	Broad	120	0.38	0.97

https://doi.org/10.1371/journal.pone.0193353.t004

possibly be associated with antibiotic prescription. However, when this factor was eliminated from the model generated during analysis, use of laboratory guidance and patient turnout still presented statistically significant relationships with antibiotic prescription rates.

Discussion

Number of prescriptions reviewed

WHO recommends at least 30 prescriptions to be reviewed per prescriber in a typical drug use investigation study [15]. However, this study reviewed 30,096 prescriptions from 56 prescribers

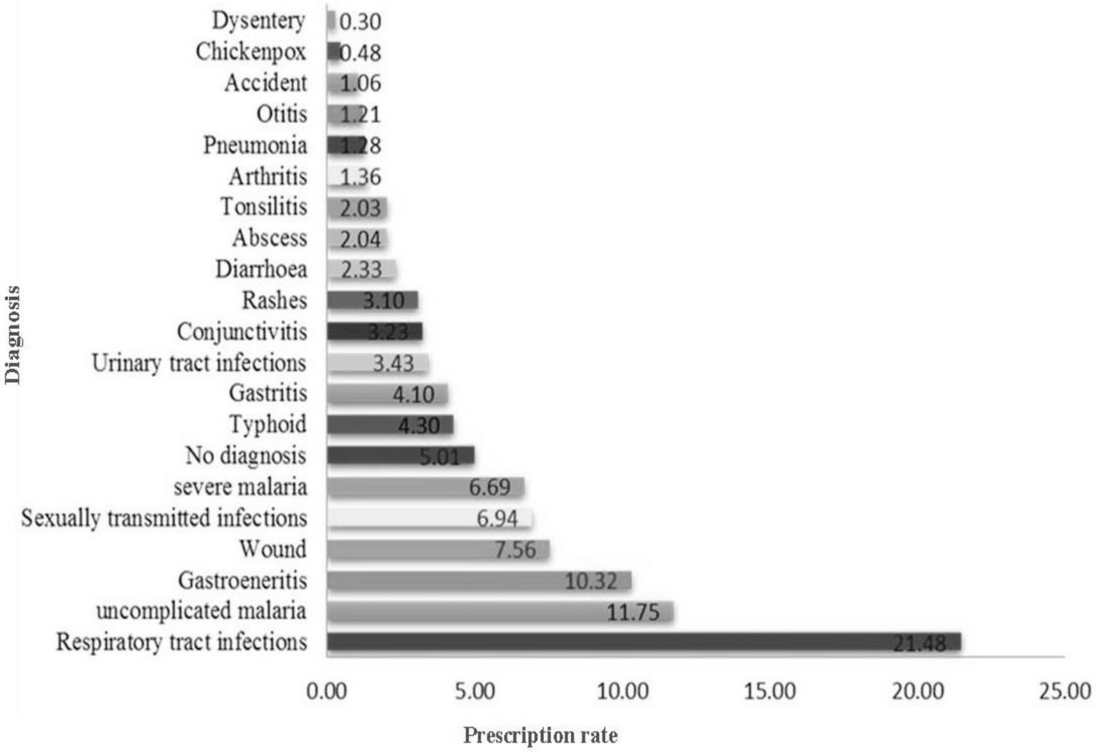

Fig 5. Common indications for antibiotic prescription.

https://doi.org/10.1371/journal.pone.0193353.g005

Table 5. Clinical and socio-demographic predictors of antibiotic prescription.

Predictor	Number	Antibiotic prescription rate	Antibiotic combination utilization (prescription pattern)		
			One	Two	More than two
**Professional level**					
Med Ass	1	25.3	279	28	10
Nurse Ass	27	39.06	171.41	18.89	1.63
Brevete N	4	24.08	147.25	16.75	0
HND	1	43.3	74	3	0
Midwife	2	37.95	124.5	14	1
SRN	14	37.08	177.93	23.71	1.57
BSN	2	40.90	153	24	0.5
GP	5	29.24	195.6	50.2	6.4
		F = -0.907, P = 0.369	***H = 4.639, P = 0.704***	***H = 9.383, P = 0.226***	***H = 11.291, P = 0.126***
**Type of facility**					
Public	39	37.83	174.77	27.67	2.23
Confessional	15	33.85	133	9.67	0.60
Private	1	25.48	279	28	10
Community	1	43.78	504	15	6
		F = 0.99, P = 0.921	***H = 3.063, P = 0.382***	***H = 12.095, P = 0.007***	***H = 10.378, P = 0.160***
**Longevity**					
<5	21	34.87	126.57	18.29	2.19
5–9	11	41.1	154.91	26.36	1.64
10–14	8	30.95	172.00	14.11	1.56
15–19	6	39.28	202.8	37	3.6
≥20	10	36.06	267	28.1	1.5
		F = 03.90, P = 0.699	***H = 10.436, P = 0.034***	***H = 1.367, P = 0.850***	***H = 2.123, P = 0.713***
**Use of Treatment guidelines**					
Yes	48	34.71	151.79	21.08	1.90
No	8	37.26	288.50	31.88	2.50
		F = 1.343, P = 0.186	***H = 2.922, P = 0.087***	***H = 1.486, P = 0.223***	***H = 2.831, P = 0.92***

https://doi.org/10.1371/journal.pone.0193353.t005

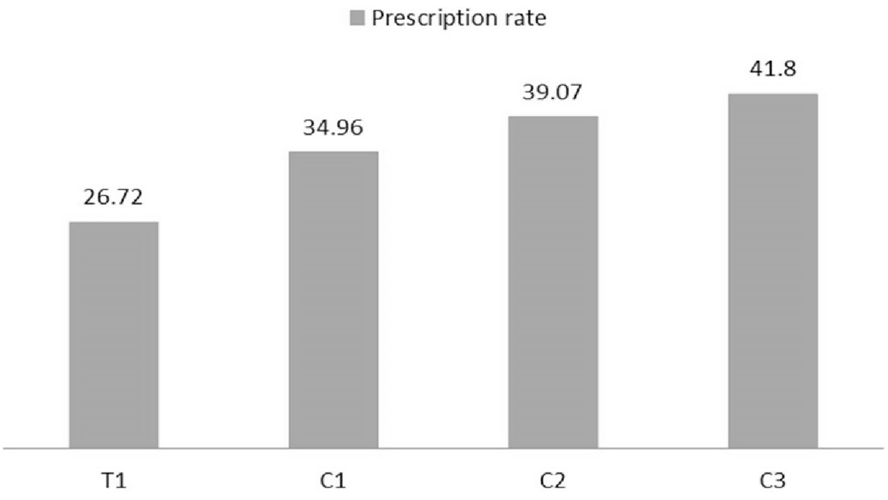

Fig 6. Antibiotic prescription rates in different performance based financing classification of facilities.

https://doi.org/10.1371/journal.pone.0193353.g006

(an average of 537.4 prescriptions per prescriber) within the periods of April 2014-April 2015. This was to obtain a holistic and true representation of antibiotic prescribing behaviors of primary health care personnel in KE and KW health districts, and roll out the chances of some prescribing patterns being missed out.

Patients prescribed antibiotics in primary health facilities in our study where from the ages 8 months to >90 years (Table 2). The highest antibiotic exposure was in patients ≤ 10 years (44%) followed by those 21–30 years (40%) while patients 81–90 years (10%) had the least prescriptions for antibiotics (Table 2). Dong *et al.* [39] reported the highest antibiotic exposure in the pediatric population compared to other age groups in Western China. However, contrary to our study in which more females (38%) than males (35%) received antibiotics, Carneiro *et al.* [40] and Shankar *et al.*, [41] reported a higher exposure in males than females in Brazil and Western Nepal respectively. We observed a significant difference in antibiotic exposure with respect to age (F = 3.40, P = 0.005) and gender (F = 8.94, P = 0.000) among patients who were prescribed antibiotics.

Overall antibiotic prescription rate

An overall antibiotic prescribing rate of 36.71% was observed in primary health care facilities within the study (Table 3). This shows a high consumption of antibiotics in the study area. Our antibiotic prescription rate was similar to the 34% observed in Malawi [9]. However, our prescribing rate was low compared to the findings in Sudan 54.3%% [42], Western China 48.43% [39], Bahrain 45.8 [43], Uzbekistan 57% [44], Nigeria 56% [25], 45.9% in the African region [45], eastern Ethiopia 82.5% [46] and in primary health care centers in the WHO African region 46.8% [47]. Our percentage of patients prescribed antibiotics was higher than 29.5% reported in Western Nepal [41] and 26.2% in Bahrain [48]. These studies investigated antibiotic prescribing patterns within a shorter duration of time (10 days and 3 months respectively) compared to our study duration of one year. Antibiotic prescribing rate in our study exceeded the WHO standard of 20–25.4% [49] but our average number of antibiotics per encounter (1.14) was within the WHO limit of 1.6–1.8 [49], showing that antibiotic combination during prescription was not common in study area.

Antibiotic category pattern

Fourteen different types of antibiotics all with broad-spectrum of activity were prescribed in study area. Prescription of broad spectrum antibiotics is a common phenomenon reported in similar studies [39–41,50]. With the use of broad spectrum antibiotics, there is no need for pathogen isolation and identification. Primary care facilities studied lack culture facilities, hence culture and antibiotic sensitivity testing is never performed. Antibiotic prescription is thus empiric, hence the tendency of prescribing only broad spectrum agents in our study area. Amoxicillin (29.29%) was the most frequently prescribed, followed by cotrimoxazole (19.08%) and metronidazole (15.59%). The majority of our study participants who were prescribed antibiotics were less than 10 years old. Children account for majority of respiratory tract infections which are treated mostly with amoxicillin [51]. The prevalence of HIV in Cameroon [52] with associated opportunistic infections that require antibiotics for treatment such as cotrimoxazole and metronidazole could possibly account for these high prescription rates in this study. In a similar study in Botswana, amoxicillin and metronidazole were the most frequently prescribed antibiotics. HIV, gynecological and sexually transmitted infections were identified as possible reasons for their use [53]. Similar antibiotics have been prescribed in other primary care settings [39–41,46,54]; however, these studies reported more antibiotics prescribed than observed in our study. In a similar study in Buea, Cameroon [14], the cephalosporines were most

frequently used antibiotic. This study was conducted in a secondary care facility where prescribers are mainly medical doctors and handle complicated and referral cases from primary care facilities. Our study focused on primary care facilities whose drug options are limited to the EDL and manage primary complications in the communities. All drugs prescribed (100%) were in the WHO list of essential medications [55]. Thirteen of the fourteen antibiotics (92.8%) were in EDL of MoH [56]. Spectinomycin was the only drug prescribed that was not in MoH list though it is present in the WHO list. However, it was the least prescribed antibiotic in our study (0.04%). 96.8% of health care facilities had a copy of the EDL. In a similar study in eastern Ethiopia [46], only 75% (6/8) of the health facilities had a copy of the essential drug list. Our percentage antibiotic prescription from the EDL was higher than 67.1% reported in Nepal [41], 88% recently reported in the WHO African region [47] and 89% in Africa [45]. The Nepal study was conducted in a tertiary care facility where the use of essential drug list is usually more limited as the necessity to use drugs not covered in the essential drug list may arise. Our findings show a high utilization of the EDL in primary care settings in our study area.

Increase in antibiotic prescribing by generic name constitutes one of the primordial indicators of prescribing of low cost antibiotics in the WHO indicator guide. In our study, 98.36% prescriptions were for generic drugs (Table 3). This was lower than the 100% recommended by WHO for prescription by generic name [49] but higher than reported in Buea, Cameroon [14], Bahrain [43], and the Africa region [45,47] where much prescribing was by brand names. Our results are similar to 97% reported in eastern Ethiopia [46] and 99.8% reported in health centers in Cambodia [57]. Generic versus brand name antibiotics have shown no differences in efficacy [58]. This is because generic prescribing reduces the chances of drug duplication as patients without knowledge, purchase and use the same drug from different prescribers when one prescriber uses brand name and another generic name or when both use different brand names. However, there exist discrepancies in knowledge and opinion over the use of generic or brand name medicines during prescribing [59]. Treatment with generic antibiotics has been shown to cause worse safe and effectiveness outcomes [60]. Thus generic prescribing as observed in our study site shows a high availability of standard medicines as generics and should be promoted.

Respiratory tract infections (21.27%) were the main diagnosis for which antibiotics were prescribed (Fig 5). Similar studies elsewhere [26,39,40,51,61,62] have reported RTIs as the most common indications for antibiotic prescription. Prognostic uncertainty and diagnostic complexity [63] of RTIs have been reported to influence antibiotic prescription decisions on RTIs. Most RTIs are of viral origin, self-limiting and do not require antibiotics for treatment [1]. However, they account for majority of antibiotics prescribed in primary health care facilities facilitating the development of antibiotic resistance [64]. We also observed antibiotics prescribed for uncomplicated malaria (11.42%), severe malaria (6.30%) and in situations with unknown indication-'No diagnosis' (5.76%). Following the present guidelines for the management of uncomplicated and severe malaria stipulated by the MoH Cameroon, no antibiotic should be administered as treatment for either condition [65]. Also, conditions with no definite diagnosis that were subject to antibiotic prescription demonstrate clear proof of antibiotic misuse which can potentially lead to increased antibiotic resistance [1] thus increasing the necessity to use more expensive antibiotics to treat life threatening infections caused by resistant bacteria in the future. Similar to a study in Uganda [66] in which majority of prescribers were lower cadre professionals (nursing aide, nursing assistant and enrolled nurses), the majority of prescribers in our study (42.8%) were Nurse Assistants whose level of training is inadequate for them to prescribe [67]. These Nurse Assistants may have been responsible for the wrong prescriptions observed. As antibiotic prescription may vary with season as a result

of seasonal variation in infectious disease prevalence, our study reviewed prescriptions for one year period which covered all the seasons.

Associated factors of antibiotic prescription

Drug availability in health facilities, socio-economic status of patients in public health facilities, in-service education of prescribers in private health facilities were identified as factors that influence drug prescription in Warri Nigeria [25]. In Malaysia, type of infection ($P < 0.001$), age of patients ($P = 0.000$) and source of payment of prescribers ($P = 0.000$) were identified as possible associated factors of antibiotic prescribing [26]. Among the possible associated factors of antibiotic prescription investigated in present study, we observed a significant relationship with use of laboratory results to guide prescription ($P = 0.000$), Performance Based Financing ($P = 0.006$) and patient turnout ($P = 0.002$) (Table 5). Among these, patient turn out has been reported by previous studies [16,17,68]. Other factors reported by these studies included prescriber's qualification, experience, source and method of updating knowledge. These were not investigated in our study. In their study on antibiotic prescribing on febrile patients in Zambia, Ndhlovu et al. [62] reported testing positive for malaria or receiving a malaria diagnosis to be associated with reduced antibiotic prescribing while testing negative, not being tested or diagnosis with upper respiratory tract to have higher rates of antibiotic prescribing. After a detailed investigation of individual associated factor strengths of factors studied, Performance Based Financing (PBF) was identified as a moderating/interacting variable for other predictors with a significant consecutive increase in prescription rates from T1, C1, C2 to C3 health facilities (Fig 6). PBF is a health service finance programme that remunerates (financially) health care facilities according to the quality and quantity of care they deliver to the population. Health care facilities subject to this programme were categorized into four different groups; T1 facilities, whose services are constantly evaluated using a quality assessment checklist and they are paid according to their productivity; C1 facilities, whose services are also constantly evaluated using quality assessment checklist but they are paid according to the productivity of T1 facilities, C2 facilities who only benefit from quality assessment checks but no financial reward and C3 who neither benefit from quality assessment checks nor financial rewards. C3's carryout their routine activities as always without any influence from the PBF programme. Therefore, lack of an antibiotic prescription regulatory agency such as PBF explains why C3 facilities presented with highest antibiotic prescription rate compared to C2, C1 and T1 health facilities that are subject to quality assessment from PBF.

Limitations of study

This was a clinic based study which did not consider self-medication, hence an underestimation of antibiotic use in study site. We did not collect data on patient recovery hence treatment outcome could not be determined. We did not pay attention to co-prescribed drugs but took note only of the number co-prescribed with antibiotics.

Conclusion

Our study shows a high adherence to the essential drug list, high prescription by generic name, low prescribing of antibiotics compared to primary care facilities in other African countries. Despite this there is misuse of antibiotics in primary health care facilities especially in the management of indications such as uncomplicated and severe malaria that do not require antibiotic interventions in their standard management guides and also in situations with unknown indication. Amoxicillin and Metronidazole were the commonly used antibiotics in primary health care facilities and variations in antibiotic prescribing rates and patterns were associated

with use of laboratory guidance prior to prescription, patient turnout and Performance Based Financing facility classification. PBF was seen to moderate antibiotic prescribing and should be extended to include all health facilities in study area and in other parts of the country. Periodic reviews of antibiotic susceptibility patterns of commonly used antibiotic should be done, alongside organization of refresher courses on proper disease diagnosis and treatment especially with antibiotics.

Supporting information

S1 Text. Questionnaire administered to primary health care personnel prescribing antibiotics in Kumbo East and Kumbo West health districts, North West region, Cameroon. (DOCX)

Author Contributions

Conceptualization: Elvis Dzelamonyuy Chem, Damian Nota Anong, Jane-Francis K. T. Akoachere.

Formal analysis: Elvis Dzelamonyuy Chem, Jane-Francis K. T. Akoachere.

Investigation: Elvis Dzelamonyuy Chem.

Supervision: Damian Nota Anong, Jane-Francis K. T. Akoachere.

Writing – original draft: Elvis Dzelamonyuy Chem, Jane-Francis K. T. Akoachere.

Writing – review & editing: Elvis Dzelamonyuy Chem, Jane-Francis K. T. Akoachere.

References

1. Llor C, Bjerrum L. Antimicrobial resistance: risk associated with antibiotic overuse and initiatives to reduce the problem. Therapeutic advances in drug safety. 2014; 5:229–41 https://doi.org/10.1177/2042098614554919 PMID: 25436105
2. Goossens H, Ferech M, Vander Stichele R, Elseviers M; ESAC Project Group. Outpatient antibiotic use in Europe and association with resistance: a cross-national database study. Lancet. 2005; 365:579–87. https://doi.org/10.1016/S0140-6736(05)17907-0 PMID: 15708101
3. Finch R. Innovation, drugs and diagnostics. J Antimicrob Chemother. 2007; 60 (1):79–82.
4. Gandra S, Barter DM, Laxminarayan R. Economic burden of antibiotic resistance: how much do we really know? Clin Microbiol Infect. 2014; 20(10):973–80. https://doi.org/10.1111/1469-0691.12798 PMID: 25273968
5. World Health Organization. Antimicrobial resistance. Fact sheet No 194. Updated September 2016. www.who.int/mediacenter/factsheet/fs194/en/
6. Fitchett JR, Atun R. Antimicrobial resistance: opportunity for Europe to establish global leadership. The Lancet Infectious Diseases. 2016; 16(4): 388–389 https://doi.org/10.1016/S1473-3099(15)00410-7 PMID: 26708523
7. Smith R, Coast J. Economic burden of antimicrobial resistance: why it is more serious than current studies suggest. Technical Report. London School of Hygiene & Tropical Medicine, London. 2012; https://doi.org/10.17037/PUBS.00639028
8. Apisarnthanarak A, Kiratisin P, Saifon P, Kitphati R, Dejsirilert S, Mundy LM. Clinical and molecular epidemiology of community-onset, extended-spectrum beta-lactamase-producing Escherichia coli infections in Thailand: a case-case-control study. Am J Infect Control. 2007; 35(9):606–12. https://doi.org/10.1016/j.ajic.2007.05.008 PMID: 17980240
9. Hogerzeil HV, Buno D, Ross-Degnan D, Laing RO, Ofori-Adjei D, Santoso B, et al. Field tests for rational drug use in twelve developing countries. *Lancet*. 1993; 342: 1408–10 PMID: 7901689
10. Gebeyehu E, Bantie L, Azage M. Inappropriate Use of Antibiotics and Its Associated Factors among Urban and Rural Communities of Bahir Dar City Administration, Northwest Ethiopia. PloS one. 2015; 10(9).

11. Holloway K, van Dijk L. WHO: The World Medicines Situation 2011. Rational use of medicines. 2011; 3rd edition, Geneva. 22pp
12. Dyar OJ, Beović B, Vlahović-Palčevski V, Verheij T, Pulcini C. How can we improve antibiotic prescribing in primary care? Expert review of anti-infective therapy. 2016; 14:403–13 https://doi.org/10.1586/14787210.2016.1151353 PMID: 26853235
13. Lloyd W, Mayer R, Chin TL Antibiotic Prescribing Policy. Royal United Hospital Bath. National Health Service, Wiltshire, 2013; pp. 2–4.
14. Mbam LA, Monekosso GL, Asongalem E A. Indications and patterns of prescription of antibiotics at the Buea Regional Hospital of Cameroon. J Health Sci Dis. 2015; 6 (1): 1–7.
15. World Health Organization. How to Investigate Drug Use in Health Facilities: Selected Drug Use Indicators. EDM Research series No. 007. WHO, Geneva Switzerland. WHO/DAP/93.1. 1993
16. Steinman MA, Landerfield CS, Gonzales R. Predictprs of broad-spectrum antibiotic prescribing for acute respiratory tract infections in adult primary care. JAMA 2003; 289:719–25 PMID: 12585950
17. Bharathiraja R, Sivakumar S, Chelliah L, Saradha S Mangayarkarasi S. Factor's affecting antibiotic prescribing pattern in pediatric practice. Indian J Pediatr.2005; 72 (6):1–5.
18. Huang AM, Newton D Kunapuli A. Antimicrobial stewardship team intervention in adult patients with bacteremia and candidemia. Clin Infect Dis.2005; 57(9):1237–1245.
19. Akkerman A, Kuyvenhoven M, Van der Wouden J, Verheij T. Prescribing antibiotics for respiratory tract infections by GPs: management and prescriber characteristics. Br J Gen Pract. 2005; 55: 114–8. PMID: 15720932
20. Cadieux G, Tamblyn R, Dauphinee D, Libman M. Predictors of inappropriate prescribing among primary care physicians. CMAJ, 2007; 177(8):877–83 https://doi.org/10.1503/cmaj.070151 PMID: 17923655
21. Charan N, Chandra DS, Adiveni T, Sundresh NJ, Kumar BA, Padmini P, et al. A prospective study: Factors affecting antibiotic prescribing pattern in surgery wards in RMMCH. J Biomed Pharm Res. 2013; 2 (3):71–73.
22. Kotwani A, Wattal C, Katewa S, Joshi P Holloway K. Factors Influencing Primary Care Physicians to Prescribe Antibiotics in India. Fam Pract. 2010; 27:684–690. https://doi.org/10.1093/fampra/cmq059 PMID: 20660529
23. Dekker AR, Verheij TJ, Velden AW. Inappropriate antibiotic prescription for respiratory tract indications: most prominent in adult patients. Fam Pract. 2015; 32(4): 401–407 https://doi.org/10.1093/fampra/cmv019 PMID: 25911505
24. Ritabul Salami MR, Hassali MA, Alrasheedy AA, Saleem F, Faridah Aryani MY, Godman B. Physicians' knowledge, perceptions, and behaviour towards antibiotic prescribing: A systematic review of the literature. Expert Rev AntiInfect Ther. 2015; 13(5):665–680
25. Erah PO, Olumide GO Okhamafe AO. Prescribing practices in two health care facilities in Warri, Southern Nigeria: A comparative study. Trop J Pharm Res.2003; 2 (1): 175–182.
26. Lim Y, Sivasampu S Hwong A, Sim B Chandrasekaran S. Prescribing patterns and factors influencing the choice of antibiotics in upper respiratory tract infections. Clinical Research Center Findings, Ministry of Health Malaysia, 2012; pp 1–2.
27. Rudasingwaa M., Soetersb R. and Basenyac O. The effect of performance-based financing on maternal healthcare use in Burundi: a two-wave pooled cross-sectional analysis. *Global health action*. 2017; 10: 1327241 https://doi.org/10.1080/16549716.2017.1327241 PMID: 28617216
28. World Bank. Performance Based Financing in Cameroon: An innovative approach to health system strengthening. RBFHEALTH. Online. Available at: https://www.rbfhealth.org/resource/performance-based-financing-cameroon-innovative-approach-health-system-strengthening, 2014. (Accessed 23/01/2018)
29. Chung A, Perera R, Brueggemann AB, Elamin AE, Elamin AE, Harnden A, et al. Effect of antibiotic prescribing on antibiotic resistance in individual children in primary care: prospective cohort study. BMJ 2007; 335:429 https://doi.org/10.1136/bmj.39274.647465.BE PMID: 17656505
30. Costelloe C, Metcalfe C, Lovering A, Mant D, Hay AD. Effect of antibiotic prescribing in primary care on antimicrobial resistance in individual patients: systematic review and meta-analysis. BMJ, 2010; 340: c2096 https://doi.org/10.1136/bmj.c2096 PMID: 20483949
31. Van der Velden AW, Bell J, Sessa A, Duerden M, Altiner A. Sore throat: effective communication delivers improved diagnosis, enhanced self-care and more rational use of antibiotics. Int J Clin Pract Suppl. 2013; (180):10–6 https://doi.org/10.1111/ijcp.12336 PMID: 24238425
32. Mainous AG III, Everett CJ, Post RE, Diaz VA, Huston WJ. Availability of antibiotic for purchase without prescription on the internet. Annals of Family Medicine, 2009; 7(5):431–435. https://doi.org/10.1370/afm.999 PMID: 19752471

33. Davey P, Brown E, Charani E, Fenelon L, Gould IM, Holmes A, et al. Interventions to improve antibiotic prescribing practices for hospital inpatients. Cochrane Database Syst Rev. 2013; 4:CD003543. https://doi.org/10.1002/14651858.CD003543.pub3
34. Donkor ES, Tetteh-Quarcoo PB, Nartey P, Agyeman IO. Self-medication practices with antibiotics among tertiary level students in Accra, Ghana: a cross-sectional study. Int J Environ Res Public Health. 20125; 9(10):3519–29. https://doi.org/10.3390/ijerph9103519 PMID: 23202760
35. Akoachere JTK, Suylika Y, Njom HA, Esemu NS. Etiologic profile and antimicrobial susceptibility of community-acquired urinary tract infection in two Cameroonian towns. BMC Res Notes, 2012; 5:219 https://doi.org/10.1186/1756-0500-5-219 PMID: 22564344
36. Fusi-Ngwa CK, Vincent KP. Antimicrobial resistance in Dschang, Cameroon. Ann Trop Med Pub Health, 2013; 6(4): 446–451
37. Jubulis J, Dionne K, Osterhout G, Ayuk L, Awasom C, Achu P, et al. Mycobacterium tuberculosis resistance in pulmonary TB patients in Cameroon: a phenotypic susceptibility assay. Int J Tuberc Lung Dis. 2015; 19 (7):823–7. https://doi.org/10.5588/ijtld.14.0527 PMID: 26056109
38. Consortium AEDES/IRESCO. Performance Based financing Implementation Manual. North West Region of Cameroon. 147pp www.fbrcameroun.org/cside/contents/docs/Procedure_Manual.pdf
39. Dong L, Yan H, Wang D. Antibiotic prescribing patterns in village health clinics across 10 provinces of Western China. J Antimicrob Chemother. 2008; 62:410–415 https://doi.org/10.1093/jac/dkn153 PMID: 18400805
40. Carneiro M, Ferraz T, Bueno M, Koch BE, Foresti C, Lena VF, et al. Antibiotic prescription in a teaching hospital: a brief assessment. Rev Assoc Med Bras. 2011; 57(4):414–417 PMID: 21876924
41. Shankar RP, Partha P, Shenoy NK, Easow JM, Brah,adathan KN. Prescribing patterns of antibiotics and sensitivity patterns of common microorganisms in the Internal Medicine ward of a teaching hospital in Western Nepal: a prospective study. Ann Clin Microbiol Antimicrob. 2003; 2:7 https://doi.org/10.1186/1476-0711-2-7 PMID: 12904265
42. Mahmoud RK, Kheder SI, Ali HM. Prescribing rationality in Khartoum state, Sudan: An update. Sudan Med Monitor. 2014; 9(2):61–66
43. Otoom S, Culligan K, Al-Assoomi B, Al-Ansari T. Analysis of drug prescription in primary health care centers in Bahrain. Eastern Mediterr Health J. 2010; 16 (5): 511–515
44. Pavin M, Nurghozin T, Hafner G, Yusufy F, Liang R. Prescribing practices of rural primary health care physicians in Uzbekistan. Trop Med Int Health. 2003; 8 (2): 182–190 PMID: 12581446
45. Holloway KA, Ivanovska V, Wagner AK, Vialle-Valentine C, Ross-Degnan D. Have we improved use of medicines in developing and transitional countries and do we know how to? Two decades of evidence. Tropical Med Int Health. 2013, 18(6):656–664
46. Bilal AI, Osman DE, Mulugeta A. Assessment of medicines use pattern using World Health Organization's prescribing, patient care and health facility indicators in selected health facilities in eastern Ethiopia. BMC Health Serv Res. 16:144 https://doi.org/10.1186/s12913-016-1414-6 PMID: 27106610
47. Ofori-Asenso R, Brhlikova P, Pollock A. Prescribing indicators at primary health care centers within the WHO African region: a systematic analysis (1995–2015). BMC Public Health. 16:724 https://doi.org/10.1186/s12889-016-3428-8 PMID: 27545670
48. Naseeb TA, Nasser MA. Drug prescribing indicators in primary health care centers in Bahrain. Saudi Med J. 2005; 26(9):1436–8. PMID: 16155664
49. Isah AO, Quick LJ, Mabadeje AFB, Santoso B, Hogerzeil H, Ross-Degnan A. The development of reference values for the WHO health facility core prescribing indicators. West Afr J Pharmacol Drug Res, 2001; 18(1&2): 6–11
50. d'Obrenan JB, Verheij TJM, Numans EM, van der Velden AW. Antibiotic use in Dutch primary care: relation between diagnosis, consultation and treatment. J Antimicrob Chemother. 2014; 69 (6): 1701–1707. https://doi.org/10.1093/jac/dku005 PMID: 24508898
51. Williams BG, Gouws E, Boschi-Pinto C, Bryce J, Dye C. Estimates of world-wide distribution of child deaths from acute respiratory infections. Lancet Infectious Diseases. 2002; 2 (1):25–26. PMID: 11892493
52. Joined United Nations program on HIV and AIDS. Country Cameroon. Updated 2017, http://www.unaids.org/en/regionscountries/countries/cameroon accessed 14/12/2017.
53. Mashalla Y, Setlhare V, Massele A, Sepako E, Tiroyakgosi C, Kgatlwane J, et al. Assessment of prescribing practices at the primary healthcare facilities in Botswana with an emphasis on antibiotics: Findings and implications. Int J Clin Pract. 2017;e13042. https://doi.org/10.1111/ijcp.13042
54. Petersen I, Hayward AC. Antibacterial prescribing in primary care. J Antimicrob Chemother, 2007; 60 (Suppl 1):i43–i4750.

55. WHO Model List of Essential Medicines. http://www.who.int/medicines/publications/essentialmedicines/en/

56. Cameroon's Essential Drug List. Liste nationale des medicaments essentiels-Cameroun

57. Chareonkul C, Khum VI, Boonshuyar C. Rational drug use in Cambodia: study of three pilot health centers in Kampong horn Province, South East Asian. J Trop Med Public Health, 2002; 33: 418–424

58. Veronin M. Should we have concerns with generic versus brand antimicrobial drugs? A review of issues. JPHSR 2011; 2:135–50

59. Fadare JO, Adeoti AO, Desalu OO, Enwere OO, Makusidi AM, Ogunleye O, et al. The prescribing of generic medicines in Nigeria: knowledge, perceptions and attitudes of physicians. Expert Review of Pharmacoeconomics and outcome research. 2016; 16 (5): 639–650.

60. Piovani D, Clavenna A, Cartabia M, Bortolotti A, Fortino I, Merlino L, et al. Comparing recurrent antibiotic prescriptions in children treated with a brand name or a generic formulation. Pharmacoepidemiol Drug Saf. 2015; 24(2):121–8. https://doi.org/10.1002/pds.3654 PMID: 24910387

61. Horwood J, Cabral C, Hay AD, Ingram J. Primary care clinician antibiotic prescribing decisions in consultations for children with RTIs: a qualitative interview study. Br J Gen Pract, 2016; https://doi.org/10.3399/bjgp16X683821

62. Ndhlovu M, Nkhama E, Miller JM, Hamer DH. Antibiotic prescribing practices for patients with fever in the transition from presumptive treatment of malaria to 'confirm and treat' in Zambia: a cross-sectional study. Trop Med Int Health. 2015; 20(12):1696–706. https://doi.org/10.1111/tmi.12591 PMID: 26311240

63. Wu Y, Yang C, Xi H, Zhang Y, Zhou Z, Hu Y. Prescription of antibacterial agents for acute upper respiratory tract infections in Beijing, 2010–2012. Eur J Clin Pharmacol. 2016; 72:359–364 https://doi.org/10.1007/s00228-015-1997-6 PMID: 26706249

64. National Institute for Health and Clinical Excellence (UK). Respiratory Tract Infections—Antibiotic Prescribing. Prescribing of Antibiotics for Self-Limiting Respiratory Tract Infections in Adults and Children in Primary Care. NICE Clinical Guidelines. 2008 No. 69; London.

65. National Malaria Control Program Guidelines for the management of malaria in Cameroon. Ministry of Public Health. 2015: p 22–36.

66. Mbonye AK, Buregyeya E, Rutebemberwa E, Clarke SE, Lal S, Hansen KS, et al. Prescription for antibiotics at drug shops and strategies to improve quality of care and patient safety: a cross-sectional survey in the private sector in Uganda. BMJ Open 2016; 6:e010632 https://doi.org/10.1136/bmjopen-2015-010632 PMID: 26980439

67. Department of human resources. Revised curriculum for training of nursing assistants. Ministry of Public Health Cameroon. Mbalmayo. 2009: p 3–6.

68. Gjelstad S, Strand J, Dalen I, Fetveit A, Strøm H,Lindbæk M. Do general practitioners' consultation rates influence their prescribing patterns of antibiotics for acute respiratory tract infections? J Antimicrob Chemother. 2011; 66: 2425–33. https://doi.org/10.1093/jac/dkr295 PMID: 21784782
